# Genome-wide identification and expression analysis of the R2R3-MYB gene family in tobacco (*Nicotiana tabacum* L.)

**DOI:** 10.1186/s12864-022-08658-7

**Published:** 2022-06-09

**Authors:** Jiahan Yang, Binghui Zhang, Gang Gu, Jiazheng Yuan, Shaojun Shen, Liao Jin, Zhiqiang Lin, Jianfeng Lin, Xiaofang Xie

**Affiliations:** 1 College of Life Sciences, Fujian Agriculture & Forestry University, Fuzhou, China; 2 Institute of Tobacco Science, Fujian Provincial Tobacco Company, Fuzhou, China; 3 Department of Biological and Forensic Sciences, Fayetteville State University, Fayetteville, NC 28301 USA; 4 Longyan Company of Fujian Tobacco Corporation, Longyan, 364000 China; 5 Yanping Branch of Nanping Tobacco Company, Nanping, China

**Keywords:** *Nicotiana tobacum* L., R2R3-MYB transcription factors, Phylogenetic analysis, Stress response, Gene expression

## Abstract

**Background:**

The R2R3-MYB transcription factor is one of the largest gene families in plants and involved in the regulation of plant development, hormone signal transduction, biotic and abiotic stresses. Tobacco is one of the most important model plants. Therefore, it will be of great significance to investigate the *R2R3-MYB* gene family and their expression patterns under abiotic stress and senescence in tobacco.

**Results:**

A total of 174 *R2R3-MYB* genes were identified from tobacco (*Nicotiana tabacum* L.) genome and were divided into 24 subgroups based on phylogenetic analysis. Gene structure (exon/intron) and protein motifs were especially conserved among the *NtR2R3-MYB* genes, especially members within the same subgroup. The *NtR2R3-MYB* genes were distributed on 24 tobacco chromosomes. Analysis of gene duplication events obtained 3 pairs of tandem duplication genes and 62 pairs of segmental duplication genes, suggesting that segmental duplications is the major pattern for *R2R3-MYB* gene family expansion in tobacco. *Cis*-regulatory elements of the *NtR2R3-MYB* promoters were involved in cellular development, phytohormones, environmental stress and photoresponsive. Expression profile analysis showed that *NtR2R3-MYB* genes were widely expressed in different maturity tobacco leaves, and however, the expression patterns of different members appeared to be diverse. The qRT-PCR analysis of 15 *NtR2R3-MYBs* confirmed their differential expression under different abiotic stresses (cold, salt and drought), and notably, *NtMYB46* was significantly up-regulated under three treatments.

**Conclusions:**

In summary, a genome-wide identification, evolutionary and expression analysis of *R2R3-MYB* gene family in tobacco were conducted. Our results provided a solid foundation for further biological functional study of *NtR2R3-MYB* genes in tobacco.

**Supplementary Information:**

The online version contains supplementary material available at 10.1186/s12864-022-08658-7.

## Background

MYB transcription factor is one of the largest members of the plant transcription factor family [1]. MYB proteins share a highly conserved DNA-binding domain (MYB), mostly located at the N-terminus of the protein. The MYB domains are characterized by one to four incomplete tandem repeat (R) structures (termed R1, R2, R3, R4). Each repeat is composed of 50 ~ 53 conserved amino acid residues and forms three α-helices. The second and third helices can be folded to form a helix-turn-helix (HTH) structure, which is involved in DNA binding [2]. Three regularly spaced tryptophan residues (or other hydrophobic residues) form a hydrophobic core in the three-dimensional structure center of HTH, which is important for maintaining the configuration of HTH [3]. Sometimes, the tryptophan residues are replaced by other amino acids, such as aromatic amino acids and hydrophobic amino acids, especially in R3 domains. The C-terminal of MYB transcription factor usually contains a transcriptional activation region rich in acidic amino acids, which is responsible for multiple protein regulatory activities. Based on the number and types of MYB repeats, the MYB family is subdivided into four major groups, namely 4R-MYB, 3R-MYB/R1R2R3-MYB, 1R-MYB/MYB-related, 2R-MYB/R2R3-MYB [4].

R2R3-MYB transcription factor is predominantly present in plants, which contains R2 and R3 domains. It was reported that the *R2R3-MYB* genes probably evolved from the loss of the R1 repeat in the *R1R2R3-MYB* gene or from the duplication of the R1 repeat in *1R-MYB* gene [5, 6]. *R2R3-MYB* gene family is widely involved in the regulation of various biological processes, including plant growth and development, hormone signaling, primary and secondary metabolism [7–9]. For example, *AtMYB106*, *AtMYB16* and *AtMYB17* are involved in the regulation of trichome branching, petal epidermal cell morphogenesis and early inflorescence development, respectively [10–12]. The biosynthesis of flavonoids is directly or indirectly controlled by three genes (*P*, *C1*, *Pl*) encoding *R2R3-MYB* domain in maize [1, 13, 14]. *MYB7* gene in *Actinidia deliciosa* positively regulated the biosynthesis of carotenoids and chlorophyll [15]. Knockdown the expression of *MYB305* of the ornamental tobacco which contains a conserved R2R3-MYB DNA binding domain resulted in the decrease expression of related genes in nectarins and flavonoid biosynthetic [16]. In addition, most *R2R3-MYB* genes have also been suggested to regulate plant responses to biotic and abiotic stress conditions. For instance, overexpression of *AtMYB75* in *Arabidopsis* can increase secondary metabolites (anthocyanins and flavonols), which can protect against pests [17]. *OsMYB6* gene of rice as a stress-responsive factor which plays the role as a positive regulator in response to drought and salt stress resistance [18]. Increasing the expression of *NtMYB4a* may promote anthocyanin accumulation, and thereby increase antioxidant capability and tolerance to low temperature of tobacco plant [19]. Tobacco *NtMYB12* overexpression adapts to low Pi stress environment via regulating the contents of flavonol and phosphorus [20].

Tobacco is one of the most important model plants [21]. Like other plants, tobacco is often subjected to stress such as low temperature, salt, strong light, drought and other stresses, which leads to the reduction of production. Leaf senescence is an important production process with a positive and orderly process accompanied by the changes of leaf color, cell structure, biochemical metabolism and gene expression level, along with a series of degradations, which is related to secondary metabolites, such as flavonoids, carotenoids, chlorophyll, etc. [22, 23]. The regulatory role of *R2R3-MYB* genes in abiotic stress response, plant growth and development has been assessed in several studies [12, 18, 24]. The investigation of the *R2R3-MYB* gene family and their expression patterns under various stresses including abiotic stress and senescence in tobacco is of great significance for the study of plant physiology and development.

In this study, a comprehensive investigation of *R2R3-MYB* gene family, including gene structures, chromosomal localization, phylogenetic relationship, motif composition, duplication events and *cis*-element compositions was performed using the current tobacco genome sequence data. Moreover, the gene expression profile in different senescence stages of tobacco leaves and expression pattern under various abiotic stresses such as cold, salt, and drought treatments were analyzed. The objectives of this study were to systematically analyze the sequence structures of tobacco *R2R3-MYB* gene family and explore the evolutionary relationship of *R2R3-MYB* gene family in plant, and thereby, reveal the expression regulation of the *R2R3-MYB* gene family members under various stresses or adversity condition of senescence. The information derived from this study lay a foundation for further functional investigation on the *R2R3-MYB* gene family in tobacco.

## Results

### Characterization and distribution of *R2R3-MYB* genes in tobacco genome

In this study, a total of 174 *NtR2R3-MYB* genes were identified in tobacco and were renamed from *NtMYB1* to *NtMYB174* (Table 1). The information of these *NtR2R3-MYB* genes and their corresponding proteins are showed in Table 1 and Additional file 1: Table S1, namely, gene ID, location, number of exons, protein length (aa), molecular weight (MW), theoretical isoelectric point (pI) and subcellular location. The protein lengths varied greatly from 192aa (NtMYB37, NtMYB39) to 505aa (NtMYB96). The molecular weights ranged from 22,003.55 Da (NtMYB37) to 55,710.67 Da (NtMYB96), and the theoretical isoelectric point (pI) ranged from 4.80 (NtMYB56) to 9.77 (NtMYB3). Subcellular localization prediction indicated that the majority NtR2R3-MYB members were located in the nucleus. In addition, the chromosome positions of some *NtR2R3-MYB* genes could not yet be defined due to the incomplete sequencing of tobacco genome.

**Table 1:** The *R2R3-MYB* family genes in *Nicotiana tabacum* L

Gene Name	Gene ID	Location	Exon	Protein length (aa)	MW (Da)	pI	Subcellular location
*NtMYB1*	Nitab4.5_0003163g0090.1	Nitab4.5_0003163	2	279	31,833.62	6.10	Nuclear
*NtMYB2*	Nitab4.5_0001730g0050.1	Nitab4.5_0001730	2	280	31,950.76	6.08	Nuclear
*NtMYB3*	Nitab4.5_0000673g0080.1	Nt15	3	231	26,853.27	9.77	Nuclear
*NtMYB4*	Nitab4.5_0000109g0340.1	Nt04	2	297	34,408.15	9.10	Nuclear
*NtMYB5*	Nitab4.5_0004437g0020.1	Nitab4.5_0004437	3	270	30,067.05	9.05	Nuclear
*NtMYB6*	Nitab4.5_0002728g0020.1	Nt23	2	299	34,523.17	8.98	Nuclear
*NtMYB7*	Nitab4.5_0006880g0040.1	Nitab4.5_0006880	2	298	34,220.92	9.10	Nuclear
*NtMYB8*	Nitab4.5_0017504g0010.1	Nitab4.5_0017504	3	215	24,922.58	8.99	Nuclear
*NtMYB9*	Nitab4.5_0000189g0200.1	Nitab4.5_0000189	3	387	43,320.24	6.41	Nuclear
*NtMYB10*	Nitab4.5_0000303g0330.1	Nt22	3	331	36,890.00	9.04	Nuclear
*NtMYB11*	Nitab4.5_0012287g0020.1	Nitab4.5_0012287	3	215	24,927.65	8.83	Nuclear
*NtMYB12*	Nitab4.5_0002222g0050.1	Nt23	4	279	31,844.87	9.46	Nuclear
*NtMYB13*	Nitab4.5_0004379g0020.1	Nitab4.5_0004379	3	308	35,692.25	6.48	Nuclear
*NtMYB14*	Nitab4.5_0001007g0040.1	Nt01	3	330	37,561.96	7.10	Nuclear
*NtMYB15*	Nitab4.5_0016704g0010.1	Nitab4.5_0016704	3	277	32,076.03	5.68	Nuclear
*NtMYB16*	Nitab4.5_0000044g0310.1	Nt14	3	281	31,984.67	5.08	Nuclear
*NtMYB17*	Nitab4.5_0000247g0080.1	Nt04	2	264	29,833.73	8.96	Nuclear
*NtMYB18*	Nitab4.5_0004662g0010.1	Nitab4.5_0004662	3	281	32,028.68	5.08	Nuclear
*NtMYB19*	Nitab4.5_0000436g0250.1	Nt14	3	256	29,567.10	5.44	Nuclear
*NtMYB20*	Nitab4.5_0007938g0010.1	Nitab4.5_0007938	3	320	36,739.11	5.75	Nuclear
*NtMYB21*	Nitab4.5_0008924g0010.1	Nitab4.5_0008924	3	376	42,817.57	5.77	Nuclear
*NtMYB22*	Nitab4.5_0002250g0030.1	Nt23	2	273	31,206.27	5.48	Nuclear
*NtMYB23*	Nitab4.5_0003631g0010.1	Nt16	3	348	39,211.35	5.90	Nuclear
*NtMYB24*	Nitab4.5_0002714g0010.1	Nt21	3	320	36,558.99	5.75	Nuclear
*NtMYB25*	Nitab4.5_0005304g0030.1	Nitab4.5_0005304	4	270	30,750.81	8.95	Nuclear
*NtMYB26*	Nitab4.5_0000232g0160.1	Nt12	3	376	42,584.38	7.78	Nuclear
*NtMYB27*	Nitab4.5_0000021g0690.1	Nt04	2	358	40,501.32	6.37	Nuclear
*NtMYB28*	Nitab4.5_0000302g0200.1	Nt22	3	278	32,005.06	5.53	Nuclear
*NtMYB29*	Nitab4.5_0010133g0010.1	Nitab4.5_0010133	2	347	38,694.05	6.02	Nuclear
*NtMYB30*	Nitab4.5_0000769g0140.1	Nitab4.5_0000769	3	335	38,067.87	5.74	Nuclear
*NtMYB31*	Nitab4.5_0005512g0020.1	Nitab4.5_0005512	3	412	45,423.31	5.61	Nuclear
*NtMYB32*	Nitab4.5_0005819g0010.1	Nitab4.5_0005819	2	361	40,247.79	6.11	Nuclear
*NtMYB33*	Nitab4.5_0000568g0090.1	Nt17	3	406	44,589.22	5.41	Nuclear
*NtMYB34*	Nitab4.5_0000274g0200.1	Nt17	4	324	36,112.10	5.45	Nuclear
*NtMYB35*	Nitab4.5_0000649g0090.1	Nt17	3	377	43,062.99	5.74	Nuclear
*NtMYB36*	Nitab4.5_0002030g0030.1	Nitab4.5_0005819	4	323	35,970.08	5.34	Nuclear
*NtMYB37*	Nitab4.5_0002529g0070.1	Nt17	3	192	22,003.55	6.60	Nuclear
*NtMYB38*	Nitab4.5_0000121g0260.1	Nt24	3	346	38,617.93	6.25	Nuclear
*NtMYB39*	Nitab4.5_0002217g0020.1	Nt03	3	192	22,011.62	6.60	Nuclear
*NtMYB40*	Nitab4.5_0000634g0210.1	Nt22	2	350	39,115.28	5.94	Nuclear
*NtMYB41*	Nitab4.5_0004704g0020.1	Nitab4.5_0004704	3	339	37,732.30	5.83	Nuclear
*NtMYB42*	Nitab4.5_0000842g0120.1	Nt04	3	355	39,487.19	5.85	Nuclear
*NtMYB43*	Nitab4.5_0000141g0070.1	Nt09	3	358	40,625.05	8.66	Nuclear
*NtMYB44*	Nitab4.5_0000116g0020.1	Nt19	4	384	43,672.85	5.03	Nuclear
*NtMYB45*	Nitab4.5_0000906g0090.1	Nitab4.5_0000906	4	415	46,855.05	4.86	Nuclear
*NtMYB46*	Nitab4.5_0000889g0030.1	Nt20	3	348	38,953.29	6.19	Nuclear
*NtMYB47*	Nitab4.5_0005586g0010.1	Nt15	3	291	33,336.13	5.99	Nuclear
*NtMYB48*	Nitab4.5_0010251g0010.1	Nitab4.5_0010251	5	294	33,247.57	8.10	Nuclear
*NtMYB49*	Nitab4.5_0001186g0080.1	Nt22	2	349	38,905.01	6.00	Nuclear
*NtMYB50*	Nitab4.5_0000057g0160.1	Nt12	3	297	34,210.97	6.27	Nuclear
*NtMYB51*	Nitab4.5_0001019g0020.1	Nitab4.5_0001019	3	335	38,102.89	5.84	Nuclear
*NtMYB52*	Nitab4.5_0000083g0200.1	Nt13	3	332	37,137.29	6.92	Nuclear
*NtMYB53*	Nitab4.5_0005842g0010.1	Nitab4.5_0005842	3	322	37,158.58	7.62	Nuclear
*NtMYB54*	Nitab4.5_0001295g0260.1	Nt04	4	338	38,240.08	6.55	Nuclear
*NtMYB55*	Nitab4.5_0001041g0130.1	Nt14	3	261	30,652.74	9.44	Nuclear
*NtMYB56*	Nitab4.5_0018794g0010.1	Nitab4.5_0018794	3	275	31,595.75	4.80	Nuclear
*NtMYB57*	Nitab4.5_0005224g0050.1	Nitab4.5_0005224	3	294	33,426.74	7.80	Nuclear
*NtMYB58*	Nitab4.5_0002137g0010.1	Nitab4.5_0002137	3	464	51,655.76	5.77	Nuclear
*NtMYB59*	Nitab4.5_0008050g0020.1	Nitab4.5_0008050	3	298	33,438.64	6.20	Nuclear
*NtMYB60*	Nitab4.5_0002906g0080.1	Nt04	3	293	33,161.35	7.01	Nuclear
*NtMYB61*	Nitab4.5_0001622g0080.1	Nt01	3	322	36,081.65	7.61	Nuclear
*NtMYB62*	Nitab4.5_0002300g0110.1	Nt22	3	449	48,659.87	5.24	Nuclear
*NtMYB63*	Nitab4.5_0000387g0040.1	Nt05	3	290	32,869.09	6.79	Nuclear
*NtMYB64*	Nitab4.5_0006658g0010.1	Nt08	3	274	31,241.42	4.84	Nuclear
*NtMYB65*	Nitab4.5_0000844g0110.1	Nt17	2	360	40,537.50	6.60	Nuclear
*NtMYB66*	Nitab4.5_0000428g0150.1	Nt19	3	450	48,565.84	5.26	Nuclear
*NtMYB67*	Nitab4.5_0000564g0080.1	Nt17	3	304	34,305.37	5.93	Nuclear
*NtMYB68*	Nitab4.5_0009259g0010.1	Nitab4.5_0009259	3	485	53,842.35	5.44	Nuclear
*NtMYB69*	Nitab4.5_0000365g0050.1	Nt17	3	257	28,763.40	7.54	Nuclear
*NtMYB70*	Nitab4.5_0000614g0110.1	Nt03	2	358	40,397.36	6.11	Nuclear
*NtMYB71*	Nitab4.5_0000081g0200.1	Nt09	3	424	47,599.55	6.47	Nuclear
*NtMYB72*	Nitab4.5_0000103g0310.1	Nt04	3	276	32,220.78	5.58	Nuclear
*NtMYB73*	Nitab4.5_0006318g0060.1	Nt03	3	305	34,332.33	6.04	Nuclear
*NtMYB74*	Nitab4.5_0003511g0010.1	Nt19	3	421	47,609.76	8.01	Nuclear
*NtMYB75*	Nitab4.5_0001863g0170.1	Nt03	3	267	30,867.70	5.94	Nuclear
*NtMYB76*	Nitab4.5_0003683g0020.1	Nitab4.5_0003683	4	323	35,789.44	4.97	Nuclear
*NtMYB77*	Nitab4.5_0001081g0060.1	Nitab4.5_0001081	3	421	47,340.48	6.71	Nuclear
*NtMYB78*	Nitab4.5_0002714g0060.1	Nt21	3	247	28,371.92	8.91	Nuclear
*NtMYB79*	Nitab4.5_0001048g0070.1	Nt22	3	364	40,464.90	6.27	Nuclear
*NtMYB80*	Nitab4.5_0002721g0050.1	Nitab4.5_0002721	3	283	32,031.79	7.63	Nuclear
*NtMYB81*	Nitab4.5_0008604g0010.1	Nitab4.5_0008604	3	255	29,530.09	9.02	Nuclear
*NtMYB82*	Nitab4.5_0002352g0080.1	Nt08	3	362	40,166.57	6.27	Nuclear
*NtMYB83*	Nitab4.5_0000578g0120.1	Nt02	3	314	35,069.22	7.52	Nuclear
*NtMYB84*	Nitab4.5_0000541g0100.1	Nt07	3	422	47,691.75	8.01	Nuclear
*NtMYB85*	Nitab4.5_0004051g0020.1	Nitab4.5_0004051	3	335	38,513.93	5.58	Nuclear
*NtMYB86*	Nitab4.5_0001213g0100.1	Nt21	5	481	53,651.94	5.66	Nuclear
*NtMYB87*	Nitab4.5_0000769g0210.1	Nitab4.5_0000769	3	327	37,985.21	5.88	Nuclear
*NtMYB88*	Nitab4.5_0004482g0030.1	Nt23	2	281	31,759.71	5.18	Nuclear
*NtMYB89*	Nitab4.5_0000041g0400.1	Nt15	3	215	25,017.47	5.89	Nuclear
*NtMYB90*	Nitab4.5_0007679g0010.1	Nitab4.5_0007679	3	317	35,491.55	6.45	Nuclear
*NtMYB91*	Nitab4.5_0001318g0060.1	Nt05	3	241	27,878.46	5.54	Nuclear
*NtMYB92*	Nitab4.5_0004993g0020.1	Nt09	3	336	38,085.43	8.57	Nuclear
*NtMYB93*	Nitab4.5_0003061g0020.1	Nt08	3	245	28,208.46	8.06	Nuclear
*NtMYB94*	Nitab4.5_0007042g0010.1	Nt01	2	284	31,955.02	5.17	Nuclear
*NtMYB95*	Nitab4.5_0004551g0030.1	Nitab4.5_0004551	2	342	39,174.80	6.01	Nuclear
*NtMYB96*	Nitab4.5_0000271g0230.1	Nitab4.5_0000271	3	505	55,710.67	5.31	Nuclear
*NtMYB97*	Nitab4.5_0004193g0070.1	Nt06	3	273	31,079.67	6.31	Nuclear
*NtMYB98*	Nitab4.5_0001334g0020.1	Nt22	3	282	32,969.78	8.54	Nuclear
*NtMYB99*	Nitab4.5_0011760g0030.1	Nitab4.5_0011760	3	323	36,003.45	6.38	Nuclear
*NtMYB100*	Nitab4.5_0003242g0050.1	Nt18	3	285	32,075.69	6.83	Nuclear
*NtMYB101*	Nitab4.5_0002160g0080.1	Nitab4.5_0002160	3	307	34,335.39	7.11	Nuclear
*NtMYB102*	Nitab4.5_0000201g0150.1	Nitab4.5_0000201	3	302	34,343.53	5.02	Nuclear
*NtMYB103*	Nitab4.5_0002560g0010.1	Nitab4.5_0002560	3	235	27,290.34	6.08	Nuclear
*NtMYB104*	Nitab4.5_0000363g0320.1	Nt17	3	351	39,800.29	4.96	Nuclear
*NtMYB105*	Nitab4.5_0000225g0010.1	Nt14	3	295	34,323.28	7.23	Nuclear
*NtMYB106*	Nitab4.5_0001442g0010.1	Nitab4.5_0001442	3	337	38,358.58	8.46	Nuclear
*NtMYB107*	Nitab4.5_0001074g0040.1	Nitab4.5_0001074	3	371	42,214.45	6.16	Nuclear
*NtMYB108*	Nitab4.5_0006893g0010.1	Nt13	3	345	39,161.20	5.04	Nuclear
*NtMYB109*	Nitab4.5_0000747g0030.1	Nt09	3	283	32,046.96	5.95	Nuclear
*NtMYB110*	Nitab4.5_0006144g0030.1	Nitab4.5_0006144	3	355	40,694.11	4.82	Nuclear
*NtMYB111*	Nitab4.5_0000205g0020.1	Nt18	2	435	47,856.34	6.60	Nuclear
*NtMYB112*	Nitab4.5_0000103g0300.1	Nt04	3	267	31,067.60	5.47	Nuclear
*NtMYB113*	Nitab4.5_0001050g0010.1	Nt08	3	314	35,688.67	7.48	Nuclear
*NtMYB114*	Nitab4.5_0003781g0060.1	Nitab4.5_0003781	3	270	30,619.96	5.06	Nuclear
*NtMYB115*	Nitab4.5_0006804g0010.1	Nitab4.5_0006804	3	329	37,315.85	6.53	Nuclear
*NtMYB116*	Nitab4.5_0008289g0010.1	Nitab4.5_0008289	2	446	49,114.75	6.32	Nuclear
*NtMYB117*	Nitab4.5_0003478g0050.1	Nitab4.5_0003478	3	328	37,275.75	7.02	Nuclear
*NtMYB118*	Nitab4.5_0005004g0050.1	Nitab4.5_0005004	3	355	40,635.36	6.47	Nuclear
*NtMYB119*	Nitab4.5_0012173g0010.1	Nitab4.5_0012173	3	323	36,591.55	8.11	Nuclear
*NtMYB120*	Nitab4.5_0000610g0290.1	Nt09	3	197	23,319.31	8.98	Nuclear
*NtMYB121*	Nitab4.5_0006516g0020.1	Nitab4.5_0006516	3	258	29,584.34	8.74	Nuclear
*NtMYB122*	Nitab4.5_0000958g0010.1	Nitab4.5_0000958	3	240	27,565.39	9.54	Nuclear
*NtMYB123*	Nitab4.5_0000063g0150.1	Nt24	3	299	33,432.60	8.86	Nuclear
*NtMYB124*	Nitab4.5_0003212g0030.1	Nt08	3	238	27,567.72	5.79	Nuclear
*NtMYB125*	Nitab4.5_0003324g0040.1	Nitab4.5_0003324	3	280	32,332.22	5.69	Nuclear
*NtMYB126*	Nitab4.5_0003179g0030.1	Nitab4.5_0003179	3	221	26,376.80	8.26	Nuclear
*NtMYB127*	Nitab4.5_0000958g0020.1	Nitab4.5_0000958	3	271	31,650.18	6.34	Nuclear
*NtMYB128*	Nitab4.5_0002293g0010.1	Nt06	3	268	31,228.87	5.93	Nuclear
*NtMYB129*	Nitab4.5_0005019g0040.1	Nitab4.5_0005019	5	293	32,724.01	6.10	Plasma Membrane
*NtMYB130*	Nitab4.5_0000244g0260.1	Nt18	3	376	42,425.45	5.93	Nuclear
*NtMYB131*	Nitab4.5_0001814g0020.1	Nt11	3	311	35,910.95	6.60	Nuclear
*NtMYB132*	Nitab4.5_0000070g0110.1	Nt17	3	252	29,568.27	8.52	Nuclear
*NtMYB133*	Nitab4.5_0001163g0150.1	Nt22	3	309	35,134.18	7.42	Nuclear
*NtMYB134*	Nitab4.5_0002626g0050.1	Nt05	6	276	31,084.17	6.77	Nuclear
*NtMYB135*	Nitab4.5_0005769g0040.1	Nitab4.5_0005769	3	294	34,548.53	5.94	Nuclear
*NtMYB136*	Nitab4.5_0000080g0070.1	Nt13	3	256	29,565.26	9.11	Nuclear
*NtMYB137*	Nitab4.5_0000895g0130.1	Nt15	3	263	30,829.51	6.13	Nuclear
*NtMYB138*	Nitab4.5_0000103g0280.1	Nt04	4	224	25,692.46	9.53	Nuclear
*NtMYB139*	Nitab4.5_0000642g0100.1	Nitab4.5_0000642	3	354	39,785.35	6.10	Nuclear
*NtMYB140*	Nitab4.5_0000278g0070.1	Nt04	2	400	43,099.14	5.62	Nuclear
*NtMYB141*	Nitab4.5_0005708g0020.1	Nitab4.5_0005708	2	401	43,138.26	5.65	Nuclear
*NtMYB142*	Nitab4.5_0001373g0030.1	Nt17	2	301	33,224.05	7.11	Nuclear
*NtMYB143*	Nitab4.5_0000569g0180.1	Nt15	2	236	25,652.79	7.74	Nuclear
*NtMYB144*	Nitab4.5_0000210g0060.1	Nt23	3	315	36,121.22	6.12	Nuclear
*NtMYB145*	Nitab4.5_0006450g0010.1	Nitab4.5_0006450	3	315	36,204.08	5.43	Nuclear
*NtMYB146*	Nitab4.5_0002578g0010.1	Nt03	1	357	39,264.88	7.12	Nuclear
*NtMYB147*	Nitab4.5_0008039g0010.1	Nitab4.5_0008039	1	258	28,738.10	9.12	Nuclear
*NtMYB148*	Nitab4.5_0011004g0010.1	Nitab4.5_0011004	3	243	28,553.78	7.25	Nuclear
*NtMYB149*	Nitab4.5_0000008g0380.1	Nt02	2	364	39,638.73	5.33	Nuclear
*NtMYB150*	Nitab4.5_0005060g0020.1	Nitab4.5_0005060	1	332	36,533.55	9.17	Nuclear
*NtMYB151*	Nitab4.5_0001818g0030.1	Nt07	3	311	35,476.48	6.05	Nuclear
*NtMYB152*	Nitab4.5_0001180g0160.1	Nt04	4	257	29,799.39	9.30	Nuclear
*NtMYB153*	Nitab4.5_0001984g0040.1	Nt12	1	329	36,163.18	9.07	Nuclear
*NtMYB154*	Nitab4.5_0000068g0400.1	Nt12	1	258	28,770.10	8.86	Nuclear
*NtMYB155*	Nitab4.5_0001176g0010.1	Nt02	3	311	35,523.52	8.19	Nuclear
*NtMYB156*	Nitab4.5_0000683g0060.1	Nt06	1	255	27,976.61	6.76	Nuclear
*NtMYB157*	Nitab4.5_0000797g0020.1	Nt04	1	258	28,448.05	7.68	Nuclear
*NtMYB158*	Nitab4.5_0002152g0050.1	Nt10	2	364	39,622.70	5.39	Nuclear
*NtMYB159*	Nitab4.5_0001966g0010.1	Nitab4.5_0001966	1	290	31,772.73	6.33	Nuclear
*NtMYB160*	Nitab4.5_0005973g0020.1	Nt06	5	275	32,382.32	9.70	Nuclear
*NtMYB161*	Nitab4.5_0000514g0130.1	Nt04	3	377	43,759.81	5.91	Nuclear
*NtMYB162*	Nitab4.5_0002403g0060.1	Nt06	3	373	43,268.20	6.39	Nuclear
*NtMYB163*	Nitab4.5_0001087g0070.1	Nt16	2	316	36,177.08	9.40	Nuclear
*NtMYB164*	Nitab4.5_0006037g0010.1	Nt05	3	338	38,823.57	6.98	Nuclear
*NtMYB165*	Nitab4.5_0000174g0270.1	Nt12	2	320	36,827.87	9.54	Nuclear
*NtMYB166*	Nitab4.5_0001612g0030.1	Nt02	4	423	47,479.16	5.92	Nuclear
*NtMYB167*	Nitab4.5_0000325g0020.1	Nt02	3	435	49,747.63	6.15	Nuclear
*NtMYB168*	Nitab4.5_0000918g0020.1	Nt10	3	445	50,496.34	5.57	Nuclear
*NtMYB169*	Nitab4.5_0007287g0010.1	Nitab4.5_0007287	1	377	43,129.36	9.36	Nuclear
*NtMYB170*	Nitab4.5_0000010g0220.1	Nt13	3	442	49,473.52	6.24	Nuclear
*NtMYB171*	Nitab4.5_0003500g0020.1	Nitab4.5_0003500	3	436	49,151.29	6.90	Nuclear
*NtMYB172*	Nitab4.5_0007139g0020.1	Nitab4.5_0007139	1	361	41,265.04	9.24	Nuclear
*NtMYB173*	Nitab4.5_0007777g0020.1	Nt20	12	471	52,750.84	5.87	Nuclear
*NtMYB174*	Nitab4.5_0003062g0070.1	Nt24	12	459	51,678.72	6.34	Nuclear

**Fig. 1:**
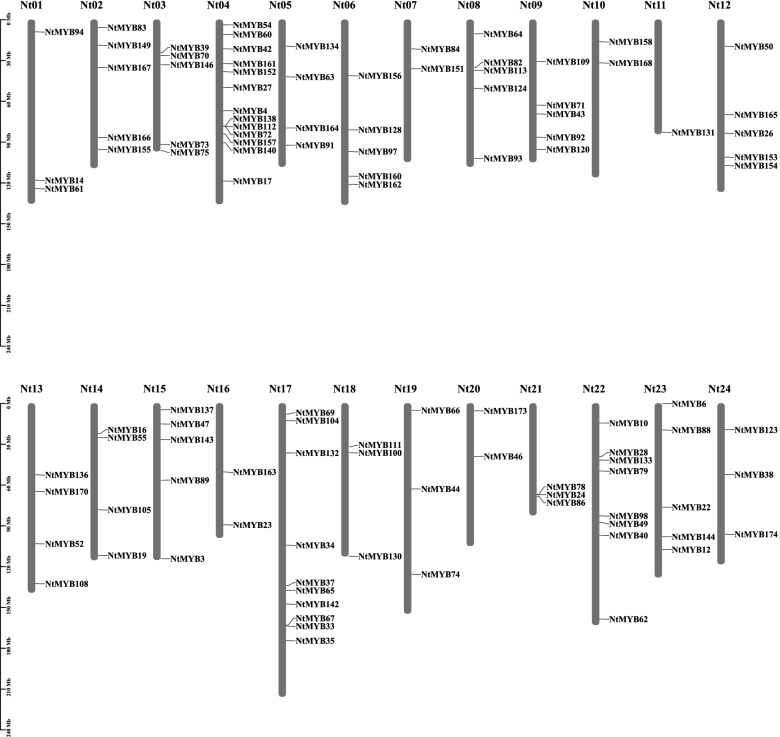
The distribution of *NtR2R3-MYB* genes on 24 chromosomes in tobacco. The scale bar of the left displays the length of tobacco chromosomes

**Fig. 2:**
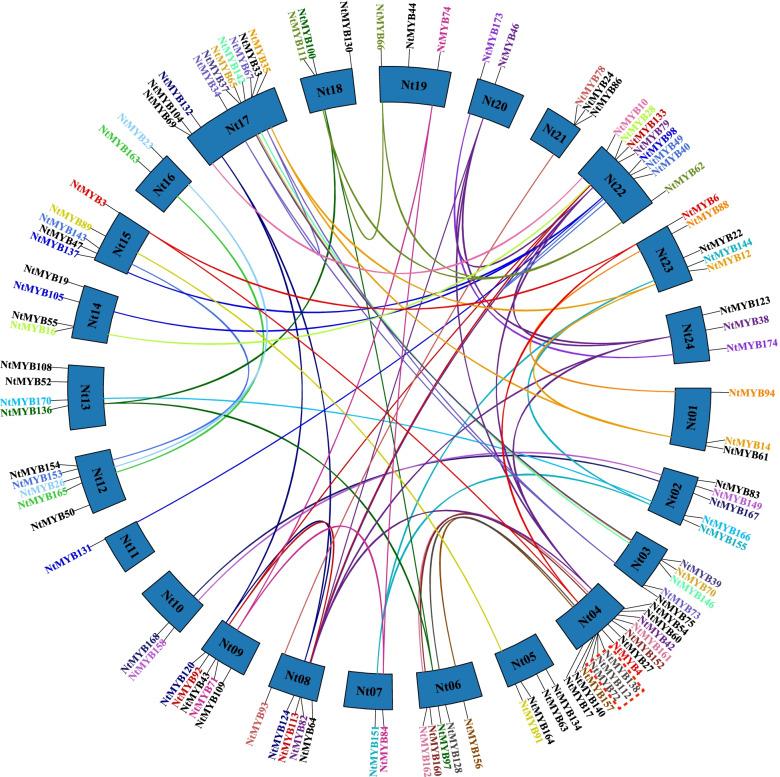
Gene duplication of *NtR2R3-MYB* genes on 24 chromosomes of tobacco genome. The segmental duplicated gene pairs are linked by different colored lines, and the tandem duplicated gene pairs of Nt04 are marked with red dashed lines. The corresponding relationships of duplicated gene are listed in Additional file 2: Table S2

A total of 107 *NtR2R3-MYB* genes were unevenly distributed on 24 chromosomes of tobacco, while 67 genes were mapped to unattributed scaffolds (Fig. 1). Chromosome 4 contained the biggest number of *NtR2R3-MYBs* (13 genes), while chromosome 17 contained 10 *NtR2R3-MYBs*, and chromosome 22 had 8 *NtR2R3-MYBs*. In contrast, chromosome 11 only contained 1 *NtR2R3-MYB* gene. In our study, 3 pairs of tandem duplication genes on chromosome 4 (*NtMYB72*/*112*, *NtMYB72*/*138*, *NtMYB112*/*138*) and 62 pairs of segmental duplication genes were identified in tobacco *R2R3-MYB* gene family (Fig. 2, Additional file 2: Table S2).

### Phylogenetics and gene structure of the *NtR2R3-MYBs*

**Fig. 3:**
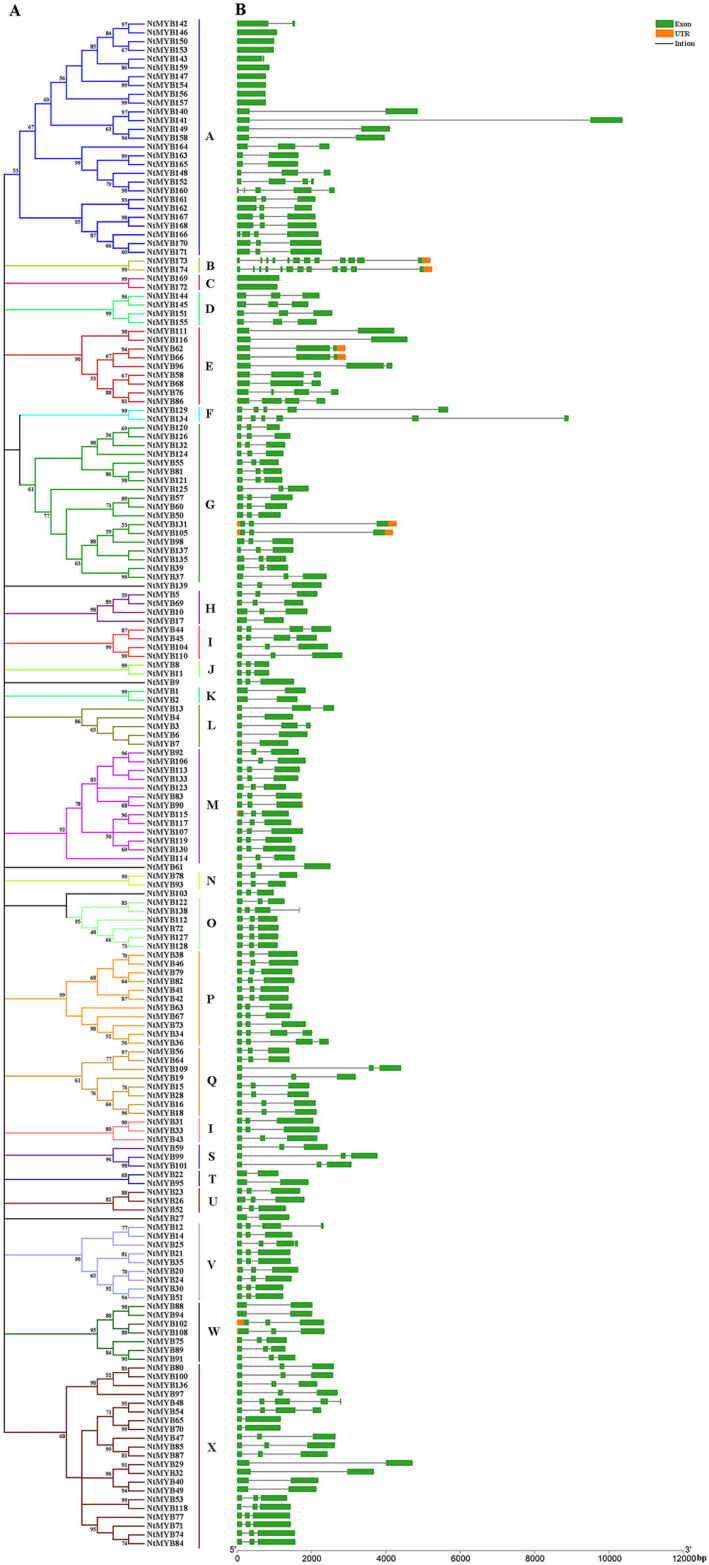
Gene structure and evolution of *R2R3-MYB* family in *Nicotiana tabacum*. **A** Phylogenetic relationships of *NtR2R3-MYBs*. Different subgroups were marked with different colors. **B** Intron-exon structure of *NtR2R3-MYB*s. Green boxes: exons; Yellow boxes: UTR; spaces between the boxes: introns. The scale bar of bottom demonstrates the length of exons and introns

To investigate the evolutionary relationships among *NtR2R3-MYB* genes, a phylogenetic tree was constructed based on the amino acid sequences of 174 *NtR2R3-MYB* genes by using the MEGA-X software (Fig. 3A). The *NtR2R3-MYBs* were classified into 24 subgroups (A to X) with at least 50% bootstrap supported on phylogenetic trees (Fig. 3A). However, 5 *NtR2R3-MYBs* (*NtMYB139*, *NtMYB9*, *NtMYB61*, *NtMYB103* and *NtMYB27*) could not be assigned to any of the 24 subgroups due to the low bootstrap values (< 50%). Among these subgroups, subgroup A (27 members) and X (21 members) were the two largest groups and these two subgroups represented more than 27% of the total *NtR2R3-MYB* members. In contrast, subfamilies B, C, F, J, K, N and T only contained two members.

Gene structure (Fig. 3B) analysis of *NtR2R3-MYBs* showed that the number of introns was varied from 0 to 11. The highest number (11) of introns was possessed by *NtMYB173* and *NtMYB174*. Notably, a great number of *R2R3-MYB* genes (119 members, 68.39%) had a conserved gene structure with two introns and three exons, while 10 *NtR2R3-MYBs* completely lacked the introns. Similar exon-intron structural patterns were found among members within the same subgroup, especially the exon number and exon length were relatively conservative (Fig. 3).

### Domain and motif analysis of the *NtR2R3-MYBs*

**Fig. 4:**
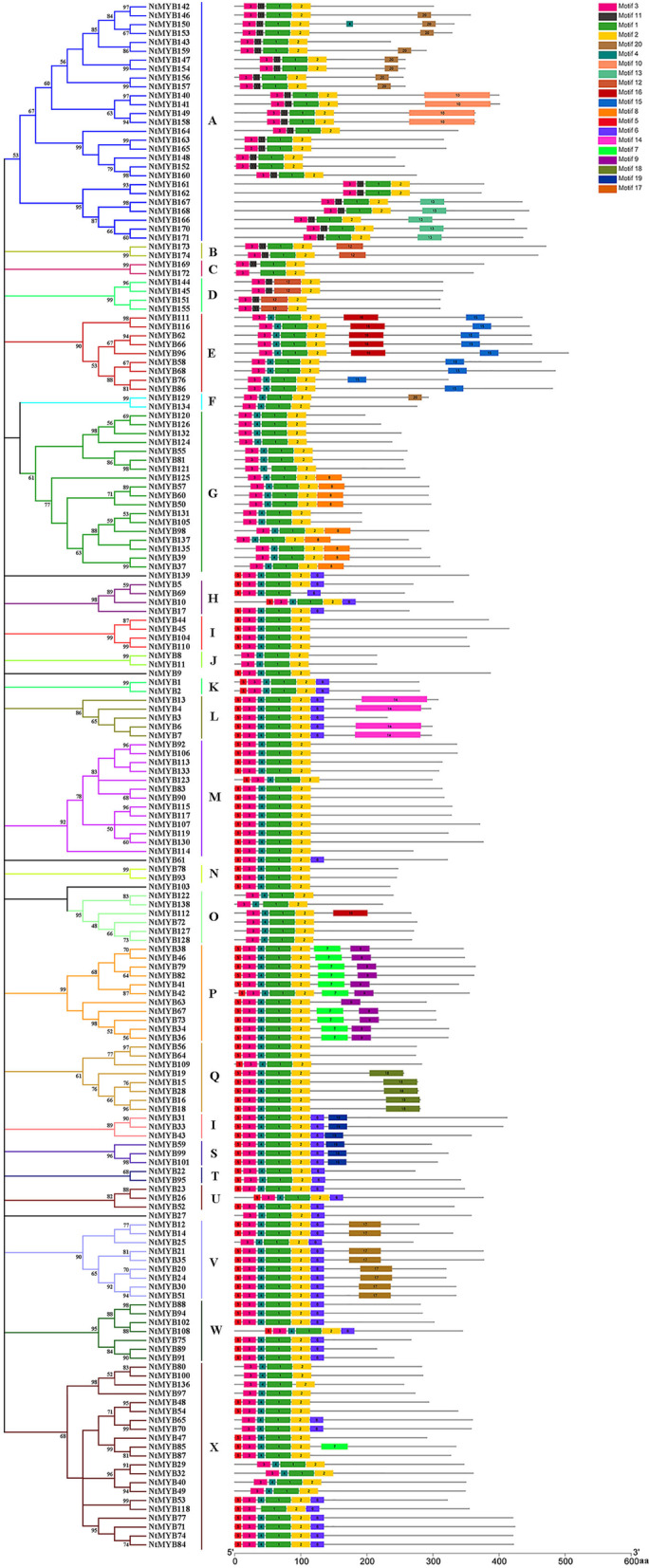
Conserved motifs for NtR2R3-MYB proteins in *Nicotiana tabacum*. Different motifs are showed with different colored boxes and numbers (1–20). The gray lines represent the non-conserved sequences. The lengths of motifs can be estimated using the scale at the bottom

A total of 20 conserved motifs were predicted for 174 NtR2R3-MYB proteins using the online MEME program (Fig. 4). The lengths and conserved sequence of each motif is listed in Additional file 3: Table S3. The motif composition and distribution were found relatively conservative among members within the same subgroup (Fig. 4). The motif1, motif2 and motif3 located in the N-terminal of the majority NtR2R3-MYB protein sequences. There were conserved tryptophan residues in these three motifs, which were related to R2 and R3 domains. The type and number of motifs were similar in same subgroup, which suggested that motif pattern might be related to the function of MYB protein. Different subgroups usually possessed specific motifs, most of which located in the C-terminal. For example: motif7 and motif9 were specific to P; motif10 and motif13 only appear in A; motif8, motif14, and motif18 was presented in G, L, and Q alone, respectively.

**Fig. 5:**
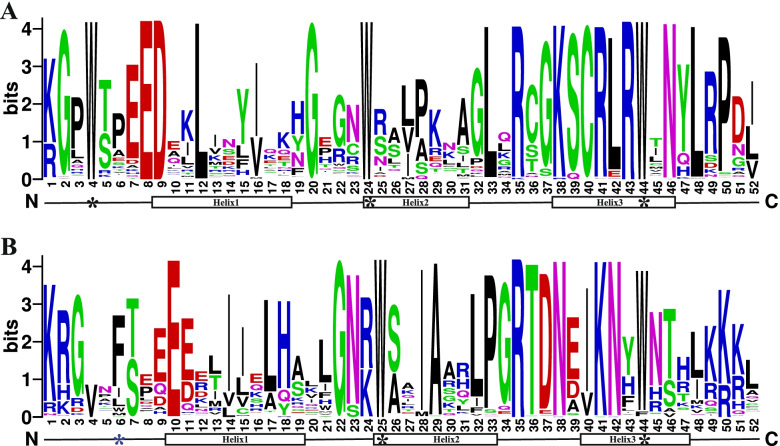
Sequence logos of the conserved R2 and R3 repeats of the NtR2R3-MYB domain. **A** Sequence logo of R2 in *NtR2R3-MYBs*. **B** Sequence logo of R3 in *NtR2R3-MYBs*. Asterisks indicate the conserved tryptophan residues (Trp) in the NtR2R3-MYB domain, and helix denote the helical structure among *NtR2R3-MYBs*

To further explore the conservative domain of the NtR2R3-MYB proteins, the multiple alignment of the 174 NtR2R3-MYB protein sequences was performed based on DNAMAN software, and the R2 and R3 sequence logos of MYB were generated by WebLogo (Fig. 5). As a result, NtR2R3-MYB members possess the typical characteristics of MYB conserved domains, and the R2 and R3 repeat of NtR2R3-MYB contain about 52 amino acid residues. The R2 repeat contains three highly conserved tryptophan residues (W), forming a hydrophobic core zin HTH structure. The first tryptophan residues (W) of R3 repeat often was replaced by a phenylalanine (F), isoleucine (I) or leucine (L) residues, whereas the second and third tryptophan residues were highly conserved, and this result was consistent with *A. thaliana* [4]. We also observed that some amino acid residues showed highly conservative, such as G-2, E-8, D-9, L-12, G-20, L-33, R-35, K-38, S-39, C-40, R-41, L-42, R-43, N-46, L-48 and P-50 in the R2 repeat and E-10, G-22, N-23, I-28, A-29, P-33, G-34, R-35, T-36, D-37, N-38, K-41 and N-42 in the R3 repeat. These highly conserved amino acid residues may be associated with conserved tryptophan residues to maintain the helix-turn-helix (HTH) structure of MYB transcription factor.

### Promoter *cis*-elements analysis of *NtR2R3-MYBs*

**Fig. 6:**
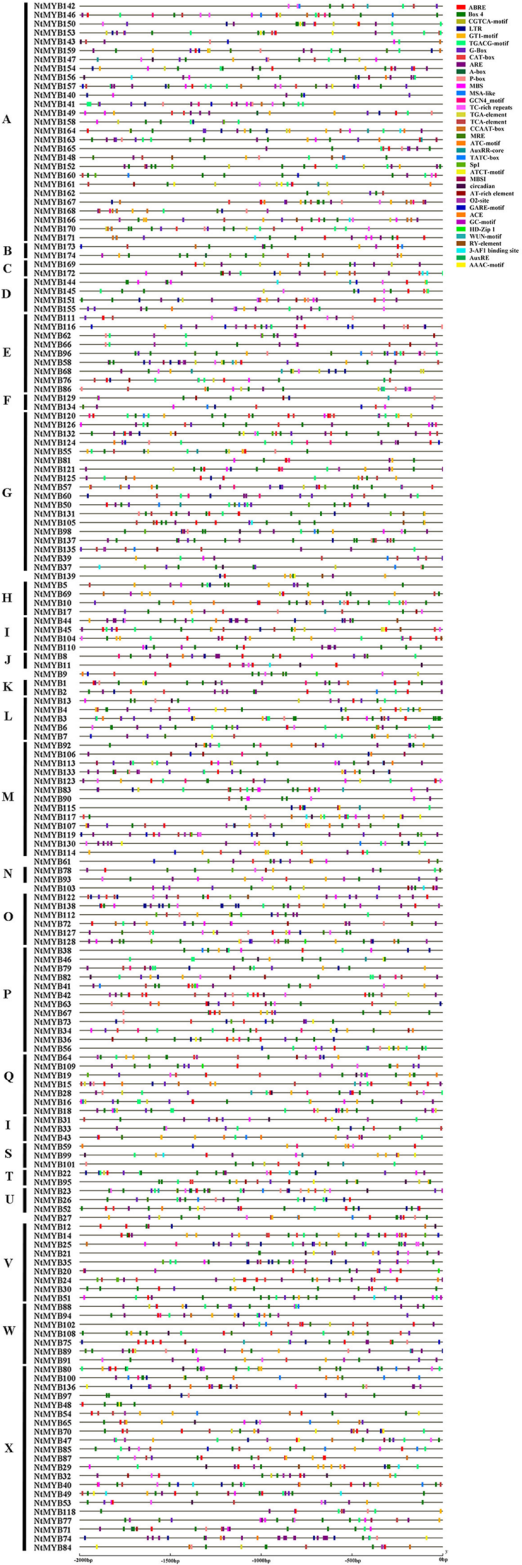
Predicted *cis*-elements in *NtR2R3-MYBs* promoters. Different shapes and colors represent the different types of *cis*-elements. Annotations of *cis*-elements were listed in Additional file 4: Table S4

Promoter *cis*-elements play critical roles in the initiation of gene expression. A total of 37 *cis*-regulatory were identified in the promoter region of *NtR2R3-MYB* genes, which could be classified into four categories elements, including cellular development, phytohormones, environmental stress and photoresponsive elements (Fig. 6, Additional file 4: Table S4). There were seven *cis*-acting elements related to cell development, such as CAT-box, MSA-like, GCN4_motif, CCAAT-box, MBSI, HD-Zip 1, and RY-element. Twelve phytohormone-responsive elements were identified, namely, CGTCA-motif, TGACG-motif, ABRE, P-box, TGA-element, TCA-element, AuxRR-core, TATC-box, GARE-motif, AuxRE, A-box and O2-site. These *cis*-elements are involved in JA/MeJA, abscisic acid, gibberellin, auxin, salicylic acid responsiveness and zein metabolism regulation. Meanwhile, the ABRE responsiveness elements were the most common in the *NtR2R3-MYB* gene promoters. In addition, ten light responsive elements were calculated, including GT1-motif, G-Box, Box 4, MRE, ATC-motif, Sp1, ATCT-motif, ACE, 3-AF1 binding site, and AAAC-motif. Almost all *NtR2R3-MYB* genes contained at least one phytohormone-responsive element in their promoter regions. There were 8 *cis*-regulatory elements that associated with response to external or environmental stresses were also present. This category includes low-temperature responsive element (LTR), anaerobic induction elements (ARE, GC-motif), drought-inducibility element (MBS), defense and stress responsive element (TC-rich repeats), circadian control element (circadian), wound-responsive element (WUN-motif), as well as AT-rich element. G-Box, Box 4, and ARE elements appear in most promoters of *NtR2R3-MYB* genes. The expression of these genes might be regulated by phytohormones, diverse light-responsiveness *cis*-elements, defense signaling transduction, and abiotic stresses during tobacco growth.

### Phylogenetic analysis of the *NtR2R3-MYB* gene family

**Fig. 7:**
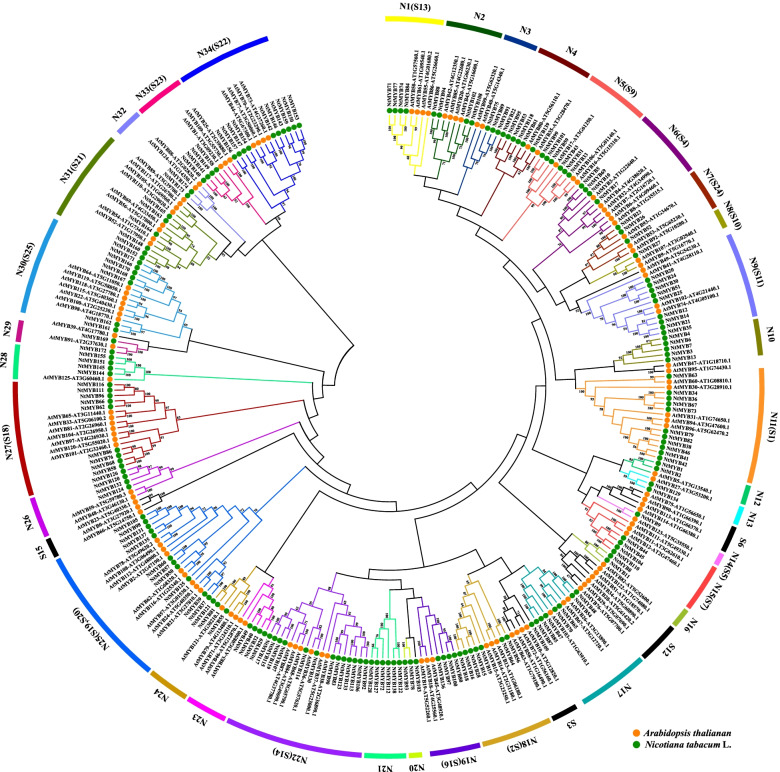
Phylogenetic tree of *Nicotiana tabacum* and *Arabidopsis thaliana R2R3-MYB* genes. The phylogenetic relationships were constructed using MEGA-X by the maximum likelihood (ML) method (1000 bootstrap replicates). The amino acid sequences of 174 NtR2R3-MYB and 126 AtR2R3-MYB proteins were used. *Arabidopsis* and tobacco are marked with yellow circles and green circles respectively

To investigate the phylogenetic relationships of the *R2R3-MYB* gene family, phylogenetic tree was generated based on the 174 tobacco R2R3-MYB protein sequences and 126 *Arabidopsis* R2R3-MYB protein sequences by using MEGA-X with maximum likelihood (ML) method (Fig. 7). According to the bootstrap values (> 50%) of the phylogenetic tree, the *R2R3-MYB* family were clustered into 38 subfamily. Among them, the R2R3-MYB members of tobacco were distributed in 34 subgroups (named N1-N34). There was no NtR2R3-MYB member distributed on the subfamily of S3, S6, S12 and S15, while three subfamilies (N10, N20, N21) only contain the R2R3-MYB members from tobacco. In addition, the NtR2R3-MYB members were mainly distributed in N9(9), N11(11), N22(13), N25(11), N27(9) and N34(10), and the number of R2R3-MYB members of tobacco were about twice to triple than that in *Arabidopsis* in these subfamilies. These results indicated that there were some common ancestors of *R2R3-MYB* genes between tobacco and *Arabidopsis*, and specific expansion and divergence also occurred after their separation during the evolution process.

### Expression changes of *NtR2R3-MYB* genes in five senescence stages of tobacco leaves

**Fig. 8:**
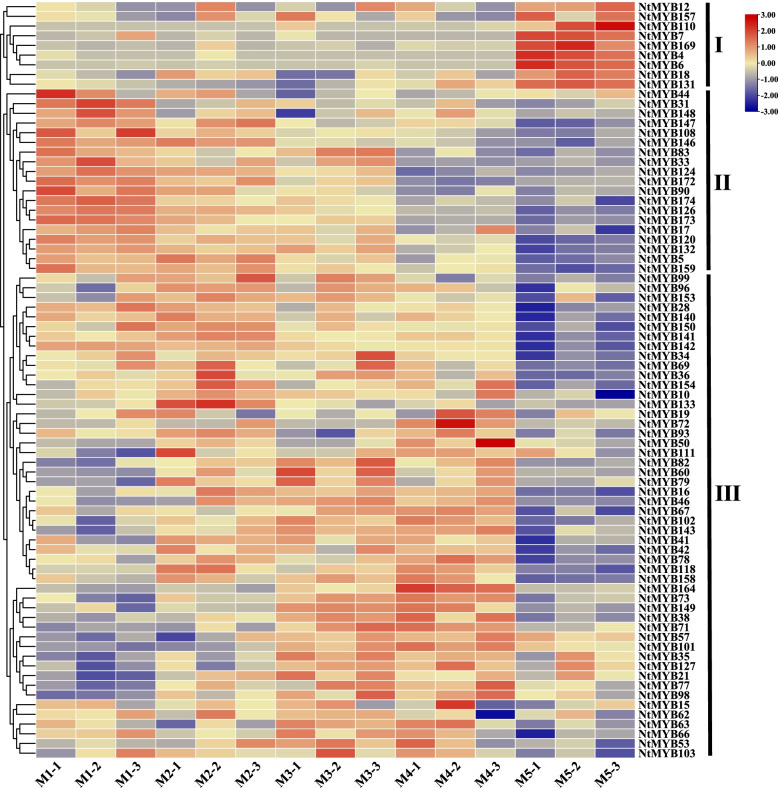
The expression of 78 *NtR2R3-MYBs* in tobacco leaves at five senescence stages. FPKM values for *NtR2R3-MYB* genes were transformed by log10. Red or blue colors represent the difference in expression levels in each sample (M1-M5), respectively

To analyze the expression pattern of *NtR2R3-MYBs* in tobacco leaves, the FPKM values of *NtR2R3-MYB* genes at five senescence stages of tobacco leaves were obtained from the transcriptome data (Additional file 5: Table S5), and *NtR2R3-MYB* genes with no expression or low expression level (FPKM < 0.5) were excluded. Finally, the expression profiles of *NtR2R3-MYB* genes of 78 *NtR2R3-MYB* genes were generated (Fig. 8). The results showed that the members of *NtR2R3-MYB* genes exhibited differential expression in tobacco leaves at different senescence stages (Fig. 8) and these 78 *NtR2R3-MYB* genes were classified into three groups (Fig. 8 I to III). A total of 9 *NtR2R3-MYB* members were including in group I, and these genes had high expression level at the M5 stage. In contrast, these genes showed relative low expression level at other four senescence stages (M1-M4). In group II, most of *NtR2R3-MYB* genes showed high expression level at the M1 stage and decreased regularly with the increase of maturity. In terms of group III, the expression level of *NtR2R3-MYB* genes showed increase first and then decreased with the increasing of the senescence degrees. The results indicated that there may be functional diversity among *NtR2R3-MYB* members during tobacco growth and development.

### Expression of *NtR2R3-MYB* genes in response to abiotic stress

**Fig. 9:**
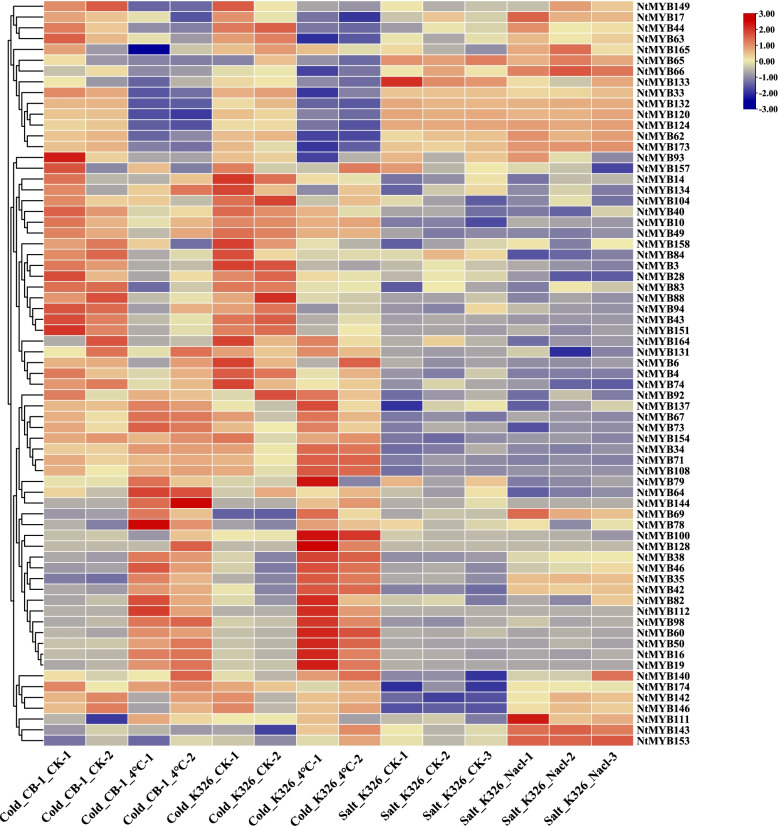
Expression of 69 *NtR2R3-MYB* genes in response to cold and slat treatments. RNA-seq data was obtained by using Genevestigator software. Expression values from RNA-seq data were log10-transformed

**Fig. 10:**
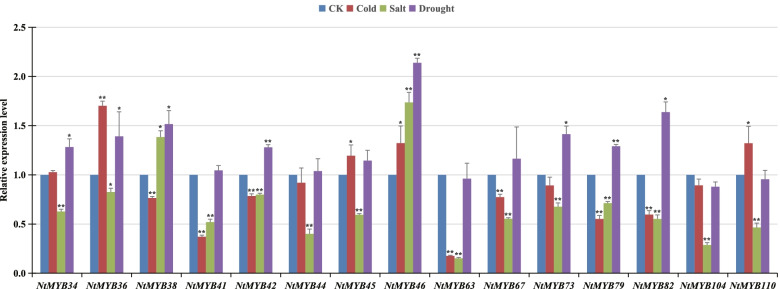
Relative expression level of 15 *NtR2R3-MYB*s in response to cold, salt, and drought treatments. Error bars are standard deviations of three biological replicates. Asterisks were used to indicate the significant degree of the expression level compared to the value of the control (*P < 0.05, **P < 0.01)

To analyze the expression pattern of *NtR2R3-MYBs* in response to abiotic stress, gene expression was investigated by using the Genevestigator tools based on transcriptome data. *NtR2R3-MYB* genes with no expression or low expression level (FPKM < 0.5) were excluded (Additional file 6: Table S6). Finally, the expression profiles of 69 *NtR2R3-MYB* genes were generated. The result showed that many genes showed significant up-regulated or down-regulated compared with the control group under cold and salt stress conditions (Fig. 9), and these genes including *NtMYB34*, *NtMYB38*, *NtMYB42*, *NtMYB44*, *NtMYB*46, *NtMYB63*, *NtMYB67*, *NtMYB73*, *NtMYB79*, *NtMYB82* and *NtMYB104* were clustered together with S1 and S7 subfamilies of *Arabidopsis*. In addition, it has been reported that the members of *Arabidopsis R2R3-MYB* gene family in S1 and S7 subgroups were related to various stress responses [25]. It is known that homologous genes in the same subgroup may have similar biological functions. To further explore the possible function of the tobacco *R2R3-MYB* genes, 15 tobacco *R2R3-MYB* genes that clustered with *Arabidopsis R2R3-MYB* gene members in S1 and S7 subgroups of the phylogenetic tree (Fig. 7) were selected for qRT-PCR analysis under cold, drought and salt stresses (Fig. 10). Compared with the control, the expression of four *NtR2R3-MYB* genes (*NtMYB36*, *NtMYB45*, *NtMYB46* and *NtMYB110*) showed significantly up-regulated and seven *NtR2R3-MYB* genes (*NtMYB38*/*41*/*42*/*63/67*/*79*/*82*) showed significantly down-regulated under cold stress. As to the salt stress, the expression levels of 13 *NtR2R3-MYB* genes showed down-regulated, except *MYB38* and *MYB46* genes. In terms of drought stress, eight *NtR2R3-MYB* genes (*NtMYB34/36/38/42/46/73/79/82*) showed significantly up-regulated. Interestingly, the expression of *NtMYB46* showed significantly up-regulated in response to all the stresses. In addition, the expression patterns of 15 genes in Fig. 9 and Fig. 10 were not completely consistent, and this phenomenon may be due to trial or sampling differences. The result implied the functional dissimilation among the tobacco *R2R3-MYB* genes.

## Discussion

*R2R3-MYB* gene family members are widely distributed in eukaryotes [26]. With the development of genome sequencing, the whole-genome analysis of *R2R3-MYB* gene family has been identified in numerous species, including 126 in *Arabidopsis thaliana*, 89 in watermelon, 110 in rice, 157 in maize, 244 in soybean, and so on [7, 14, 27–29]. In this study, 174 *R2R3-MYB* genes of tobacco were identified, the number was greater than those identified in *Arabidopsis*, maize, rice, watermelon, but less than that in soybean*.* As an allotetraploid, the genome size of *Nicotiana tabacum* is 4.5Gb, while that of *Arabidopsis*, rice, maize, and soybean is 125 Mb, 430 Mb, 2300 Mb, and 1.025Gb, respectively [30–34]. In this case, it seems that there is no direct correlation between the number of *R2R3-MYB* genes and genome size in these plants. It was reported that polyploidization and gene region-specific duplication (tandem duplication and segmental duplications) were important mechanisms for the generation and expansion of gene families in plant [35]. In our study, phylogenetic analysis found that the majority subfamilies contained the *R2R3-MYB* members both from tobacco and *Arabidopsis*, and however, a few subfamilies possessed only the *R2R3-MYB* members either from tobacco or *Arabidopsis*, suggesting that they might be derived from a common ancestor, and moreover, the *R2R3-MYB* gene family could also be undergone species specific differentiation after their separation. A total of 3 pairs of tandem duplication genes and 62 pairs of segmental duplication genes were identified in tobacco *R2R3-MYB* gene family, implying that the segmental duplication events were the main source for the expansion of *R2R3-MYB* gene family in tobacco, and this result was possible due to the allotetraploid of tobacco.

The evolution of gene family largely depends on the organization of gene structure. The varied length of nucleotide sequence among 174 *NtR2R3-MYBs* indicated the complexity in the *Nicotiana tabacum* L. genome. The molecular weight and isoelectric point values of NtR2R3-MYB proteins were also different among family members, suggesting their functional divergence. In addition, NtR2R3-MYB proteins contained 20 conserved motifs with different compositions, and their members were clustered in the same subfamily containing similar type and number of motifs, demonstrating the conservation and diversification of *R2R3-MYB* gene family of tobacco. It has been reported that the ancestor *MYB* gene has no intron, but the intron insertion event occurred in the MYB domain region under very occasional condition, and this intron pattern was kept conserved during the long evolution process [5, 36]. In our study, the majority *NtR2R3-MYB* genes possess typically splicing pattern of three exons and two introns, which exists in the conserved R2 and R3 repeats, and this result was in consistent with the previous reports in other plants [14, 37]. In addition, the NtR2R3-MYB proteins are comprised of the highly conserved MYB domain (R2 repeat and R3 repeat), R2 repeat contained a conserved LRPD motif at the C-terminal and three highly conserved tryptophan residues (W), whereas R3 repeat exist diversity at the first tryptophan residues (W), which could be substituted by other residues such as phenylalanine (F), isoleucine (I) and leucine (L). Meanwhile, substitution of amino acid occurred frequently in some sites of the MYB domain, and these regions may play important roles for the evolution and functional differentiation of tobacco R2R3-MYB protein. Similar results have been reported in other species, including *Arabidopsis*, *Zea mays* (Fig. 5) [14, 25].

The *cis*-elements of promoter play a key role in initiating gene expression. Genes with different *cis*-regulatory elements in the promoter sequences of genes may result in different expression patterns in pepper [38]. In our study, a total of 37 *cis*-elements related sequences were identified in the promoter region of *NtR2R3-MYB* genes, and among them, 7 were related to cell development, 12 engaged in phytohormone-responsive, 10 for light responsive elements, and 8 involved in stresses *cis*-regulatory elements, suggesting the different function of regulatory elements of *NtR2R3-MYB* genes. These highly diverse *cis*-regulatory elements in the promoter region of *NtR2R3-MYB* genes may also reflect the functional divergence at the transcriptional level.

*R2R3-MYB* genes have been proved to be related to biotic and abiotic stress [8]. For example, *TaMYB344*-overexpressing of wheat enhanced the tolerance of transgenic tobacco to drought, heat and high salt stress [39]. *OsMYB2* was induced by cold, salt, and dehydration stress in rice [40]. In present study, the profiling data of gene expression was used to dissect the functional roles of *NtR2R3-MYB* genes, and different expression patterns were identified among *NtR2R3-MYB* genes in response to various abiotic stresses (Fig. 9). This result indicated the functional differentiation of *NtR2R3-MYB* genes. In addition, the expression of 15 *NtR2R3-MYB* genes were analyzed by qRT-PCR in response to three abiotic stress conditions, including cold, salinity, and drought. A total of 13 *NtR2R3-MYB* genes could be significantly regulated by at least two treatments except *NtMYB44* and *NtMYB104*, implied that *NtR2R3-MYBs* may be involved in the cross-talk of different signaling pathways under stress. Generally, genes with similar structure will be clustered in the same subfamily and these genes may have similar biological functions. It was reported that two *R2R3-MYB* genes of *Arabidopsis* (*AtMYB60* and *AtMYB94*) clustered in subfamily S1 (Fig. 7) were involved in the physiological regulation under salt and drought treatments [41, 42], and therefore, the orthologous clustered in S1 may have similar function. In this study, two *NtR2R3-MYB* genes (*NtMYB38* and *NtMYB46*) which clustered with these two *R2R3-MYB* genes of *Arabidopsis* (*AtMYB60* and *AtMYB94*) in S1 subfamily showed similar response patterns under salt and drought stresses, suggesting that *NtMYB38* and *NtMYB46* may be involved in the response under salt and drought stresses, and further experiments showed be conducted to validation these functions.

Tobacco leaf senescence is often regarded as a kind of adversity, and *R2R3-MYB* genes were proved to be involved in the senescence accompany with secondary metabolites, including flavonoids, carotenoids, chlorophyll, and so on [23, 43]. In this study, diverse expression patterns were found among the *NtR2R3-MYB* genes inferring that the functional differentiation of family gene members should also be coexisting (Fig. 8). For example, *NtMYB3*/*4* and *NtMYB3/6* appeared to belong to segmental duplication genes pairs (Fig. 2, Additional file 2: Table S2), and our result showed that *NtMYB4* and *NtMYB6* expressed in all the five senescence stages, with especial high expression level in M5 stage*,* whereas *NtMYB3* had no expression in all investigated senescence stages (M1-M5) (Fig. 8). It has been reported that there are three outcomes in the evolution of duplicate genes theoretically, including nonfunctionalization, neofunctionalization and subfunctionalization [44]. Therefore, it was inferred that *NtMYB3* degenerated and lost its original function during the long evolution process. Notably, the expression level of majority members in group II including such as *NtMYB146*, *NtMYB108*, *NtMYB90*, *NtMYB159*, *NtMYB120*, *NtMYB17* (Fig. 8) were decreased precisely corresponding consistent to the increasing of the senescence degrees, and these gene may closely relate to leaf senescence and can be further developed as the measure of leaf senescence or maturity. Whether or not the differential expression of *NtR2R3-MYB* genes leads to the changes of secondary metabolites such as flavonoids, carotenoids or chlorophyll still needs to be investigated. In short, our results provide using information for their further functional exploration.

## Conclusions

In this study, a total of 174 *R2R3-MYB* genes were identified in tobacco (*Nicotiana tabacum* L.) genome and these genes were divided into 24 subfamilies. The *NtR2R3-MYB* genes were distributed randomly on 24 tobacco chromosomes. A total of 3 pairs of *NtR2R3-MYB* genes were founded to be originated from tandem duplication and 62 pairs *NtR2R3-MYB* genes were originated from segmental duplication. *Cis*-regulatory elements of the *NtR2R3-MYB* promoters were involved in cellular development, phytohormones, environmental stress and photoresponsive. The members of *NtR2R3-MYB* genes showed differential expression pattern in different maturity tobacco leaves, and differential response were also found under different abiotic stresses (cold, salt and drought) for 15 *NtR2R3-MYBs*. Our results provided valuable information for further functional study of *NtR2R3-MYB* genes in tobacco.

## Methods

### Identification of *R2R3-MYB* genes in tobacco

A total of 126 known *Arabidopsis* R2R3-MYB protein sequences were downloaded from the Arabidopsis Information Resource (TAIR, http://www.arabidopsis.org/) database [25]. These sequences were used as queries using the online tool of BLASTP (E ≤ 1e^− 5^) for the identification of R2R3-MYB family members in the tobacco genome sequences of Sol Genomics Network database (https://solgenomics.net/organism/Nicotiana_attenuata/genome) [45, 46]. The redundant protein sequences of tobacco were removed manually, and then the candidate protein sequence which contained complete MYB domains (PF000249) were confirmed as the final R2R3-MYB protein sequence based on the Conserved Domain Database (CDD) of NCBI (https://www.ncbi.nlm.nih.gov/cdd/) [47]. These final tobacco *R2R3-MYB* genes were renamed (*NtMYBs*). The features and the subcellular localization information of the NtR2R3-MYB protein sequences were analyzed by the online ExPASY tool (http://Web.ExPASY.Org/protparam/) [48] and the Softberry service platform-ProtComp 9.0 (http://linux1.softberry.com/berry.phtml), respectively.

### Gene structure and conserved motif analysis

The GFF format file of tobacco gene structure was downloaded from Solanaceae genome database (https://sol-genomics.net/) [46], and the *NtR2R3-MYB* gene structure (exon-intron) was defined using the online software Gene Structure Display Server (GSDS) (http://gsds.cbi.pku.edu.cn/) [49]. The motifs of NtR2R3-MYB protein were obtained from the online MEME program (http://meme-suite.org/, v5.1.1) [50] and following parameters were used: the minimum width, maximum width and maximum number of motifs were set to 6 bp, 100 bp and 20, respectively. The conserved NtR2R3-MYB domain was visualized using the WebLogo platform (http://weblogo.berkeley.edu/) [51]. The *cis*-regulatory elements in the promoter region (2000 bp upstream of the starting codon) of the *NtR2R3-MYBs* were searched by the online program of PlantCARE (http://bioinformatics.psb.ugent.be/webtools /plantcare/html/) [52].

### Chromosome localization and gene duplication

The physical position and chromosomal distribution information of *NtR2R3-MYB* genes were obtained by using the MapInspect software (http://mapinspect.software.informer.com/) [53]. The possible segmental duplication and tandem duplication events were defined based on the method reported by *Wang* et al. *(2010)* [54]. Both chromosomal localization and duplication events of the *NtR2R3-MYB* genes were graphic displayed using the TBtools software [55].

### Multi-sequence alignment and phylogenetic classification

To explore the evolutionary relationship of *R2R3-MYB* gene family, the full protein sequences of R2R3-MYB from *Arabidopsis* and tobacco were used for the phylogenetic tree construction. Multiple sequence alignment was performed using ClustalW program in MEGA-X software [56], and the phylogenetic tree was constructed using the maximum likelihood (ML) with 1000 bootstraps.

### *NtR2R3-MYB* genes expression analysis

The tobacco variety of Cuibi 1 (CB-1) was used in this study. To analyze the *NtR2R3-MYB* genes expression profile at different senescence stages, five senescence stages (M1, M2, M3, M4 and M5) of middle leaves (8th to 10th) judged by the appearance characteristics were collected for tests [57]. The FPKM (Fragments Per Kilobase of transcript sequence per Millions base pairs sequenced) value of the *NtR2R3-MYB* genes at five senescence stages of tobacco leaves were extracted from our recent RNA-Seq data [58]. The expression profile of *NtR2R3-MYB* genes at different senescence stages were measured by their FPKM value, and the heat map was generated using the Heatmap function of R gplots package [59]. Additionally, expression analysis of *NtR2R3-MYBs* under salt (SRP193166) [60] and cold stress (SRP097876) [61] of tobacco were performed using Genevestigator software. Genes with low expression level (FPKM < 0.5) were filtered.

### Plant treatments and quantitative real-time PCR analysis

To further decipher the expression pattern of *NtR2R3-MYB* genes in response to various abiotic, tobacco seeds were sown in sterilized mixed soil (vermiculite: humus = 1:1) under the condition of 22 °C and 16 h light/8 h dark photoperiod for 60 days [62]. The plantlets of 60 days were transplanted into a tray with a nutrient solution for 3 days in growth chamber, and then were exposed to the abiotic treatments, including cold (4 °C), drought (10% polyethylene glycol) and salt (200 mM NaCl), respectively. Untreated plantlets were used as control (CK). The samples for gene expression analysis were collected 6 h after treatment, and three biological replicates per treatment and 3 leaves for each sample from different plantlet were gathered and these samples were immediately stored at − 80 °C prior to RNA extraction.

Total RNA was extracted using the Hipure Plant RNA Mini Kit (Magen Biotech, Shanghai, China) and the cDNA was synthesized using the SMART kit (Takara) according to the manufacturer’s protocol. The qRT-PCR primers of *NtR2R3-MYB* genes were designed by online software primer3 (https://bioinfo.ut.ee/primer3-0.4.0/) [63] and were shown in Additional file 7: Table S7. Real-time quantitative RT-PCR (qRT-PCR) was performed with SYBR Green qPCR Premix (Low ROX). A total of 20 μl volume of reaction mixture for each PCR run was prepared, containing 1.5 μl cDNA, 1 × Taq SYBR Green qPCR Premix (Monad, China) and a primer pair with a concentration of 0.2 μM. The two-step thermal cycling profile used was 95 °C for 5 min, 40 cycles at 95 °C for 30 s, followed by 60 °C for 60 s. Three technical replicates were performed for each sample. The relative expression level was calculated by the 2^-ΔΔCt^ method [64].

## Supplementary Information

**Additional file 1.**

**Additional file 2.**

**Additional file 3.**

**Additional file 4.**

**Additional file 5.**

**Additional file 6.**

**Additional file 7.**

## Data Availability

The datasets generated and/or analysed during the current study are available in the the NCBI Sequence Read Archive repository, https://www.ncbi.nlm.nih.gov/sra/PRJNA772550.

## Abbreviations

HTH: Helix-turn-helix structure
MW: Molecular weight
pI: Isoelectric points
ML: Maximum likelihood
*NtR2R3-MYB*: *R2R3-MYB* gene of *Nicotiana tabacum*
FPKM: Fragments Per Kilobase of transcript sequence per Millions base pairs sequenced
GSDS: Gene Structure Display Server
qRT-PCR: Quantitative real-time PCR

## References

[1] Paz-Ares J, Ghosal D, Wienand U, Peterson PA, Saedler H (1987). The regulatory *c1* locus of *Zea mays* encodes a protein with homology to myb proto-oncogene products and with structural similarities to transcriptional activators. EMBO J.

[2] Ogata K, Kanei-Ishii C, Sasaki M, Hatanaka H, Nagadoi A, Enari M, Nakamura H, Nishimura Y, Ishii S, Sarai A (1996). The cavity in the hydrophobic core of Myb DNA-binding domain is reserved for DNA recognition and trans-activation. Nat Struct Biol.

[3] Wilkins O, Nahal H, Foong J, Provart NJ, Campbell MM (2009). Expansion and diversification of the Populus R2R3-MYB family of transcription factors. Plant Physiol.

[4] Dubos C, Stracke R, Grotewold E, Weisshaar B, Martin C, Lepiniec L (2010). MYB transcription factors in *Arabidopsis*. Trends Plant Sci.

[5] Jiang C, Gu J, Chopra S, Gu X, Peterson T (2004). Ordered origin of the typical two- and three-repeat *Myb* genes. Gene..

[6] Rosinski JA, Atchley WR (1998). Molecular evolution of the Myb family of transcription factors: evidence for polyphyletic origin. J Mol Evol.

[7] Yanhui C, Xiaoyuan Y, Kun H, Meihua L, Jigang L, Zhaofeng G, Zhiqiang L, Yunfei Z, Xiaoxiao W, Xiaoming Q, Yunping S, Li Z, Xiaohui D, Jingchu L, Xing-Wang D, Zhangliang C, Hongya G, Li-Jia Q (2006). The MYB transcription factor superfamily of *Arabidopsis*: expression analysis and phylogenetic comparison with the rice MYB family. Plant Mol Biol.

[8] Martin C, Paz-Ares J (1997). MYB transcription factors in plants. Trends Genet.

[9] Jin H, Martin C (1999). Multifunctionality and diversity within the plant MYB-gene family. Plant Mol Biol.

[10] Baumann K, Perez-Rodriguez M, Bradley D, Venail J, Bailey P, Jin H, Koes R, Roberts K, Martin C (2007). Control of cell and petal morphogenesis by R2R3-MYB transcription factors. Development..

[11] Jakoby MJ, Falkenhan D, Mader MT, Brininstool G, Wischnitzki E, Platz N, Hudson A, Hülskamp M, Larkin J, Schnittger A (2008). Transcriptional profiling of mature *Arabidopsis* trichomes reveals that *NOECK* encodes the MIXTA-like transcriptional regulator *MYB106*. Plant Physiol.

[12] Zhang Y, Cao G, Qu LJ, Gu H (2009). Characterization of *Arabidopsis* MYB transcription factor gene *AtMYB17* and its possible regulation by LEAFY and AGL15. J Genet Genomics.

[13] Cone KC, Cocciolone SM, Burr FA, Burr B (1993). Maize anthocyanin regulatory gene *pl* is a duplicate of *c1* that functions in the plant. Plant Cell.

[14] Du H, Feng BR, Yang SS, Huang YB, Tang YX (2012). The R2R3-MYB transcription factor gene family in maize. PLoS One.

[15] Ampomah-Dwamena C, Thrimawithana AH, Dejnoprat S, Lewis D, Espley RV, Allan AC (2019). A kiwifruit (*Actinidia deliciosa*) R2R3-MYB transcription factor modulates chlorophyll and carotenoid accumulation. New Phytol.

[16] Liu G, Ren G, Guirgis A, Thornburg RW (2009). The MYB305 transcription factor regulates expression of nectarin genes in the ornamental tobacco floral nectary. Plant Cell.

[17] Onkokesung N, Reichelt M, van Doorn A, Schuurink RC, van Loon JJ, Dicke M (2014). Modulation of flavonoid metabolites in *Arabidopsis thaliana* through overexpression of the MYB75 transcription factor: role of kaempferol-3,7-dirhamnoside in resistance to the specialist insect herbivore *Pieris brassicae*. J Exp Bot.

[18] Tang Y, Bao X, Zhi Y, Wu Q, Guo Y, Yin X, Zeng L, Li J, Zhang J, He W, Liu W, Wang Q, Jia C, Li Z, Liu K (2019). Overexpression of a MYB family gene, *OsMYB6*, increases drought and salinity stress tolerance in transgenic Rice. Front Plant Sci.

[19] Luo Q, Liu R, Zeng L, Wu Y, Jiang Y, Yang Q, Nie Q (2020). Isolation and molecular characterization of *NtMYB4a*, a putative transcription activation factor involved in anthocyanin synthesis in tobacco. Gene..

[20] Song Z, Luo Y, Wang W, Fan N, Wang D, Yang C, Jia H (2020). *NtMYB12* positively regulates Flavonol biosynthesis and enhances tolerance to low pi stress in *Nicotiana tabacum*. Front Plant Sci.

[21] Chen WK, Yu KJ, Liu B, Lan YB, Sun RZ, Li Q, He F, Pan QH, Duan CQ, Wang J (2017). Comparison of transcriptional expression patterns of carotenoid metabolism in 'Cabernet Sauvignon' grapes from two regions with distinct climate. J Plant Physiol.

[22] Hörtensteiner S (2006). Chlorophyll degradation during senescence. Annu Rev Plant Biol.

[23] Liu Y, Wang L, Liu H, Zhao R, Liu B, Fu Q, Zhang Y (2016). The antioxidative defense system is involved in the premature senescence in transgenic tobacco (*Nicotiana tabacum NC89*). Biol Res.

[24] Zhang Q, Zhai J, Shao L, Lin W, Peng C (2019). Accumulation of Anthocyanins: an adaptation strategy of *Mikania micrantha* to low temperature in winter. Front Plant Sci.

[25] Stracke R, Werber M, Weisshaar B (2001). The *R2R3-MYB* gene family in *Arabidopsis thaliana*. Curr Opin Plant Biol.

[26] Riechmann JL, Heard J, Martin G, Reuber L, Jiang C, Keddie J, Adam L, Pineda O, Ratcliffe OJ, Samaha RR, Creelman R, Pilgrim M, Broun P, Zhang JZ, Ghandehari D, Sherman BK, Yu G (2000). *Arabidopsis* transcription factors: genome-wide comparative analysis among eukaryotes. Science..

[27] Katiyar A, Smita S, Lenka SK, Rajwanshi R, Chinnusamy V, Bansal KC (2012). Genome-wide classification and expression analysis of MYB transcription factor families in rice and *Arabidopsis*. BMC Genomics.

[28] Du H, Yang SS, Liang Z, Feng BR, Liu L, Huang YB, Tang YX (2012). Genome-wide analysis of the MYB transcription factor superfamily in soybean. BMC Plant Biol.

[29] Wang J, Liu Y, Chen XL, Kong QS (2020). Characterization and divergence analysis of duplicated *R2R3-MYB* genes in watermelon. J Am Soc Hortic Sci.

[30] Sierro N, Battey JN, Ouadi S, Bakaher N, Bovet L, Willig A, Goepfert S, Peitsch MC, Ivanov NV (2014). The tobacco genome sequence and its comparison with those of tomato and potato. Nat Commun.

[31] Kaul S, Koo HL, Jenkins J (2000). Analysis of the genome sequence of the flowering plant *Arabidopsis thaliana*. Nature..

[32] Burr B (2002). Mapping and sequencing the rice genome. Plant Cell.

[33] Schnable PS, Ware D, Fulton RS (2009). The B73 maize genome: complexity, diversity, and dynamics. Science..

[34] Schmutz J, Cannon SB, Schlueter J (2010). Genome sequence of the palaeopolyploid soybean. Nature..

[35] Cannon SB, Mitra A, Baumgarten A, Young ND, May G (2004). The roles of segmental and tandem gene duplication in the evolution of large gene families in *Arabidopsis thaliana*. BMC Plant Biol.

[36] Fedorova L, Fedorov A (2003). Introns in gene evolution. Genetica..

[37] Jiang C, Gu X, Peterson T (2004). Identification of conserved gene structures and carboxy-terminal motifs in the *Myb* gene family of *Arabidopsis* and *Oryza sativa* L. ssp. indica. Genome Biol.

[38] Islam S, Sajib SD, Jui ZS, Arabia S, Ghosh A (2019). Genome-wide identification of glutathione S-transferase gene family in pepper, its classification, and expression profiling under different anatomical and environmental conditions. Sci Rep.

[39] Wei Q, Chen R, Wei X, Liu Y, Zhao S, Yin X, Xie T (2020). Genome-wide identification of R2R3-MYB family in wheat and functional characteristics of the abiotic stress responsive gene *TaMYB344*. BMC Genomics.

[40] Yang A, Dai X, Zhang WH (2012). A R2R3-type MYB gene, *OsMYB2*, is involved in salt, cold, and dehydration tolerance in rice. J Exp Bot.

[41] Cominelli E, Galbiati M, Vavasseur A, Conti L, Sala T, Vuylsteke M, Leonhardt N, Dellaporta SL, Tonelli C (2005). A guard-cell-specific MYB transcription factor regulates stomatal movements and plant drought tolerance. Curr Biol.

[42] Lee SB, Suh MC (2015). Cuticular wax biosynthesis is up-regulated by the MYB94 transcription factor in *Arabidopsis*. Plant Cell Physiol.

[43] Zhu F, Luo T, Liu C, Wang Y, Yang H, Yang W, Zheng L, Xiao X, Zhang M, Xu R, Xu J, Zeng Y, Xu J, Xu Q, Guo W, Larkin RM, Deng X, Cheng Y (2017). An R2R3-MYB transcription factor represses the transformation of α- and β-branch carotenoids by negatively regulating expression of *CrBCH2* and *CrNCED5* in flavedo of *Citrus reticulate*. New Phytol.

[44] Lynch M, Conery JS (2000). The evolutionary fate and consequences of duplicate genes. Science..

[45] Edwards KD, Fernandez-Pozo N, Drake-Stowe K, Humphry M, Evans AD, Bombarely A, Allen F, Hurst R, White B, Kernodle SP, Bromley JR, Sanchez-Tamburrino JP, Lewis RS, Mueller LA (2017). A reference genome for *Nicotiana tabacum* enables map-based cloning of homeologous loci implicated in nitrogen utilization efficiency. BMC Genomics.

[46] Fernandez-Pozo N, Menda N, Edwards JD, Saha S, Tecle IY, Strickler SR, Bombarely A, Fisher-York T, Pujar A, Foerster H, Yan A, Mueller LA (2015). The Sol Genomics Network (SGN)--from genotype to phenotype to breeding. Nucleic Acids Res.

[47] Marchler-Bauer A, Derbyshire MK, Gonzales NR, Lu S, Chitsaz F, Geer LY, Geer RC, He J, Gwadz M, Hurwitz DI, Lanczycki CJ, Lu F, Marchler GH, Song JS, Thanki N, Wang Z, Yamashita RA, Zhang D, Zheng C, Bryant SH (2015). CDD: NCBI's conserved domain database. Nucleic Acids Res.

[48] Wilkins MR, Gasteiger E, Bairoch A, Sanchez JC, Williams KL, Appel RD, Hochstrasser DF (1999). Protein identification and analysis tools in the ExPASy server. Methods Mol Biol.

[49] Hu B, Jin J, Guo AY, Zhang H, Luo J, Gao G (2015). GSDS 2.0: an upgraded gene feature visualization server. Bioinformatics..

[50] Bailey TL, Johnson J, Grant CE, Noble WS (2015). The MEME suite. Nucleic Acids Res.

[51] Crooks GE, Hon G, Chandonia JM, Brenner SE (2004). WebLogo: a sequence logo generator. Genome Res.

[52] Lescot M, Déhais P, Thijs G, Marchal K, Moreau Y, Van de Peer Y, Rouzé P, Rombauts S (2002). PlantCARE, a database of plant cis-acting regulatory elements and a portal to tools for in silico analysis of promoter sequences. Nucleic Acids Res.

[53] Wu GQ, Li ZQ, Cao H, Wang JL (2019). Genome-wide identification and expression analysis of the WRKY genes in sugar beet (*Beta vulgaris* L.) under alkaline stress. PeerJ..

[54] Wang L, Guo K, Li Y, Tu Y, Hu H, Wang B, Cui X, Peng L (2010). Expression profiling and integrative analysis of the CESA/CSL superfamily in rice. BMC Plant Biol.

[55] Chen C, Chen H, Zhang Y, Thomas HR, Frank MH, He Y, Xia R (2020). TBtools: an integrative toolkit developed for interactive analyses of big biological data. Mol Plant.

[56] Kumar S, Stecher G, Li M, Knyaz C, Tamura K (2018). MEGA X: molecular evolutionary genetics analysis across computing platforms. Mol Biol Evol.

[57] Qin M, Zhang B, Gu G, Yuan J, Yang X, Yang J, Xie X (2021). Genome-wide analysis of the G2-like transcription factor genes and their expression in different senescence stages of tobacco (*Nicotiana tabacum* L.). Front Genet.

[58] Zhang B, Yang J, Gu G, Jin L, Chen C, Lin Z, Song J (2021). Xie X. integrative analyses of biochemical properties and transcriptome reveal the dynamic changes in leaf senescence of tobacco (*Nicotiana tabacum* L.). Front Genet.

[59] Walter W, Sánchez-Cabo F, Ricote M (2015). GOplot: an R package for visually combining expression data with functional analysis. Bioinformatics..

[60] Xu J, Chen Q, Liu P, Jia W, Chen Z, Xu Z (2019). Integration of mRNA and miRNA analysis reveals the molecular mechanism underlying salt and alkali stress tolerance in tobacco. Int J Mol Sci.

[61] Jin J, Zhang H, Zhang J, Liu P, Chen X, Li Z, Xu Y, Lu P, Cao P (2017). Integrated transcriptomics and metabolomics analysis to characterize cold stress responses in *Nicotiana tabacum*. BMC Genomics.

[62] Song Z, Pan F, Yang C, Jia H, Jiang H, He F, Li N, Lu X, Zhang H (2019). Genome-wide identification and expression analysis of *HSP90* gene family in *Nicotiana tabacum*. BMC Genet.

[63] Untergasser A, Cutcutache I, Koressaar T, Ye J, Faircloth BC, Remm M, Rozen SG (2012). Primer3--new capabilities and interfaces. Nucleic Acids Res.

[64] Livak KJ, Schmittgen TD (2001). Analysis of relative gene expression data using real-time quantitative PCR and the 2(−Delta Delta C(T)) method. Methods..

