# Supplementary material for: Genome-wide identification and expression analysis of the R2R3-MYB gene family in tobacco (Nicotiana tabacum L.)
Source: BMC Genomics. 2022 Jun 9;23:432. doi: 10.1186/s12864-022-08658-7 (PMC9178890; doi:10.1186/s12864-022-08658-7)
Supplement: Supplementary file 3 — Additional file 3. [file 12864_2022_8658_MOESM3_ESM.docx]

**Table S3** **Sequences of 20 predicted motifs of NtR2R3-MYB proteins**

| **Motif** | **Width** | **Motif Sequence** |
| --- | --- | --- |
| 1 | 39 | RCGKSCRLRWTNYLRPDJKRGNFTEEEEELIIELHALLG |
| 2 | 29 | NRWSAIAARLPGRTDNEIKNYWNTHLKKK |
| 3 | 21 | KKGPWTPEEDEKLINYIQKHG |
| 4 | 11 | NWRALPKNAGL |
| 5 | 11 | MGRAPCCDKNG |
| 6 | 21 | LJSMGIDPVTHKPLSDLLNLS |
| 7 | 41 | SKEGSSSSSQNSISKGQWERRLQTDIHMAKQALCEALSLDK |
| 8 | 40 | AKQLKCDVNSKQFKDTLRYLWMPRLVERIQAAATTSSSQS |
| 9 | 30 | KQEPVQSSSTYASSAENIARLLQNWMKNSP |
| 10 | 100 | FGISKLLEGVFDDPLIPHRCGHGCCGAPSMEFNGSSLLGPEFVDYEELPAVSSHELAAJATDJNNIAWCKSGLENAGRLVGRTPATRGYQGSSHLTPRPF |
| 11 | 11 | PRNWSLIAEKJ |
| 12 | 41 | QRTGKSCRLRWVNKLRPNLKNGVKFSAEEERTVIELQAQFG |
| 13 | 37 | TNATPPYSDDDSPSLLTHQTCDEEMNFMQNLFGKNSL |
| 14 | 100 | SRNNSFENVTLSTTCSSNSNFEEADVENKENEVKLEEVSFFPRDLDFGKLLEGDANLDEFLIQESSYISNKYSLPMNESMLEKVYEEYLLLLSENCYLQD |
| 15 | 29 | LSPRNSGLLDALLYESQTLKASKKNSHQE |
| 16 | 53 | PDFLPINDFDIPAVEFKNLELNQQLYPPPLLEIPSNSLLDIPASSLLAQGLSS |
| 17 | 49 | NTQLQNQEGSELLLQKFQENQLLNAPMQNQLPTFQPNNQFQNQIPEIPT |
| 18 | 52 | MEGAGQELQVQFPFSSVKZENVDMLGTKLEDDMDFWYNVFIKSGDLPDLPEF |
| 19 | 29 | AANLRHMAQWESARLEAEARLVRESKLLV |
| 20 | 21 | PEFLGVMQDMIAKEVRNYMSG |
