# Supplementary material for: Genome-wide identification and expression analysis of the R2R3-MYB gene family in tobacco (Nicotiana tabacum L.)
Source: BMC Genomics. 2022 Jun 9;23:432. doi: 10.1186/s12864-022-08658-7 (PMC9178890; doi:10.1186/s12864-022-08658-7)
Supplement: Supplementary file 7 — Additional file 7. [file 12864_2022_8658_MOESM7_ESM.docx]

**Table S7 Tobacco *NtR2R3-MYB* gene-specific primers used for qRT-PCR analysis**

| Primer Name | Forward Primer (5′→3′) Sequence | Reverse Primer (5′→3′) Sequence |
| --- | --- | --- |
| NtActin | ACCTCTATGGCAACATTGTGCTCAG | CTGGGAGCCAAAGCGGTGATT |
| NtMYB34 | AGCAAGAGCTGCAGGCTAAG | GCTATTGCAGCCCATTTGTT |
| NtMYB36 | AAAGCAAAGGTGGAATGGTG | CATAACGGACAAGGGCAGTT |
| NtMYB38 | CCAACACCAGAGGGGTTAGA | CCCTCTTGGGAAAAACCATT |
| NtMYB41 | GAAGGCCACCTTGTTGTGAT | GATTCCCGGACGGAGATAAT |
| NtMYB42 | AGAGCAAGCCAAGTTTGGAA | ATCAGAAGCATCACCCAAGG |
| NtMYB44 | GGAAAGAGTTGCCGACTGAG | CCTGGTAAATGTGCCGCTAT |
| NtMYB45 | TCTAGAGAGGCCCAGAGCAG | TATTTTCTGCGTCGTTGCTG |
| NtMYB46 | AGTGAAGGGACCATGAATGC | TTCCAGCATTCTCAGGTGTG |
| NtMYB63 | GCAAAAGTTGCAGGCTAAGG | ATTGTCCGTCCTTTGTGGAA |
| NtMYB67 | GGACCATGGACTCCTGAAGA | TCTTGCTACATCTGCGCAAC |
| NtMYB73 | GGACCATGGACTCCTGAAGA | TCTTGCTACATCTGCGCAAC |
| NtMYB79 | AACATGGTCCTGGAAACTGG | CCAAGAAGAGCTTGGAGGTG |
| NtMYB82 | AGGACCATGGACACCAGAAG | TCCACGTTTAATCCCTGGAC |
| NtMYB104 | TCAAGAGAGGCAGGTGGACT | CTCAGTCGGCAACTCTTTCC |
| NtMYB110 | CGTGCAACTTTGGGTAACAG | CAGCTTGTGGCAACTTTTCA |
